# The Microalga *Chlorella vulgaris* Supplements as a Factor Increasing the Survival of Potentially Probiotic Lactic Acid Bacteria Under Environmental Stress Conditions

**DOI:** 10.1111/1758-2229.70226

**Published:** 2025-11-14

**Authors:** Patryk Adamski, Lucyna Kłębukowska

**Affiliations:** ^1^ Department of Food Microbiology, Meat Technology and Chemistry, Faculty of Food Science University of Warmia and Mazury Olsztyn Poland

**Keywords:** algae, functional foods, *lactobacillus* sp, probiotic, stress factors, superfoods, survival

## Abstract

Recent studies show that *Chlorella vulgaris* , due to its richness in nutrients, exhibits protective properties for probiotic strains of lactic acid bacteria (LAB). This creates an opportunity to produce more effective probiotics. This study aimed to evaluate the effect of two supplements of *C. vulgaris* on the growth and survival of LAB exposed to adverse stress factors (pH = 3, the presence of 0.2% and 0.5% bile salts). Survival was evaluated after 0 and 6 h of culture in the adverse stress environment. The results obtained, indicated statistically significant (*p* < 0.05) differences in the obtained growth and survival of the tested groups of LAB in the presence of *C. vulgaris* and the control sample. The study found that LAB showed the lowest survival rate in the presence of 0.5% bile salts, regardless of the culture variant. The results obtained suggest that the effect of *C. vulgaris* on the growth and survival of LAB strains depends on various factors, including the culture environment, the source of isolation of the strain and the chemical composition and nutritional content of the preparation. The results confirm the protective nature of *C. vulgaris* in the presence of high concentrations of bile salts and low pH.

## Graphical abstract

*Chlorella vulgaris* supplementation enhanced the growth and survival of lactic acid bacteria under acidic and bile stress, confirming its protective role. These findings highlight the potential of *C. vulgaris* as a functional additive to improve probiotic viability in food applications.

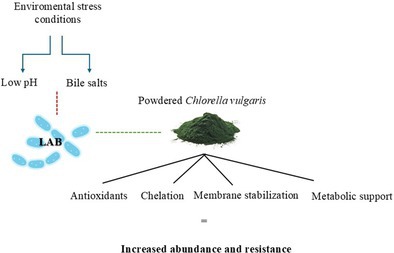

## 1 Introduction

*Chlorella vulgaris* is a green, eukaryotic microalga within the genus *Chlorella*, present on the Earth since the Precambrian period. In the early 1990s, German scientists noticed a high protein content in the cells of *C. vulgaris* and began to see it as a new source of food (Safi et al. 2014). Since then, interest in the microalgae has continued to grow. Recent studies show that *C. vulgaris* due to its richness in nutrients exhibits prebiotic properties on probiotic strains of LAB (Ścieszka and Klewicka 2020). This creates an opportunity to produce more effective probiotics or foods richer in LAB. In light of today's incoming research results, the impact of microbiota on the life and functioning of humans is enormous and still being discovered (Chen et al. 2021; Thriene and Michels 2023).

Probiotics are defined as living microorganisms that, when supplied in appropriate amounts provide health benefits to the host. The positive health effects have been proven mainly for specific probiotic strains, belonging to the following genera: *Lactobacillus*, *Bifidobacterium*, *Saccharomyces*, *Enterococcus*, *Streptococcus*, *Pediococcus*, *Leuconostoc*, *Bacillus* (Fijan 2023). A source of probiotics can be dietary supplements, pharmaceutical preparations, or food products such as yogurts, pickles, or kefirs (Putta et al. 2018; Pyo et al. 2024).

Among the features that characterize probiotic strains we distinguish functional and technological aspects. Functional features include for example, origin from the human organism, strictly defined taxonomic affiliation, lack of pathogenicity, resistance to gastric acid and bile salts when orally administered, ability to produce substances with antimicrobial activity, ability to adhere to epithelial cells, antagonism to common pathogens of the gastrointestinal tract or genitourinary tract, competition for receptors with cellular microorganisms such as *Escherichia coli* and *Salmonella Typhimurium* , and a benefit to the health of the host confirmed in clinical studies. Probiotic strains should be able to retain their properties after the technological process and a relatively long storage period (Jach et al. 2013; Kiepś and Dembczyński 2022). The credibility of the manufacturer's indicated health claims of probiotics, and their degree of safety, must be determined based on clinical studies (Ducatelle et al. 2014; Lerner et al. 2019). When studying the therapeutic effects of probiotic and food products containing probiotic strains, it is important to note that the product at the time of consumption must contain 10^6^ cells/cm^3^ of live and active cells (Sikora et al. 2020).

The survival, growth, and functionality of probiotic bacteria are strongly influenced by various environmental stress factors encountered during food production, storage, and passage through the gastrointestinal tract. These stressors can be classified as abiotic, including temperature fluctuations, pH changes, osmotic stress, oxidative stress, and exposure to bile salts, as well as biotic factors, such as competition with other microorganisms and exposure to bacteriophages (Papadimitriou et al. 2016; Sionek et al. 2024). Abiotic stresses, such as low pH or high osmolarity, can impair cell membrane integrity, enzyme activity, and metabolic processes, thereby reducing the viability of probiotics in functional foods (Liu et al. 2022; Bentahar et al. 2024). Biotic stress factors, including interspecies interactions and microbial antagonism, can further affect the persistence and colonisation ability of probiotics in complex microbial ecosystems (Bustos et al. 2025; Khoo et al. 2025). Understanding these stress responses is crucial for the design of effective probiotic formulations and for ensuring their health‐promoting properties in the final product (Papadimitriou et al. 2016; Bustos et al. 2025).

The most important carbohydrate in *C. vulgaris* , in terms of nutritional usefulness is β‐1,3‐glucan, which is a branched‐chain polysaccharide composed of β‐D‐glucose units. β‐1,3‐glucan is also, among other things, a fibre that is water‐soluble and easily fermented in the large intestine; it is an important part of the diet, aiding digestion. In addition to β‐1,3‐glucan, *C. vulgaris* cells contain almost all the vitamins (A, B1, B2, B6, B12, C, E, biotin, pantothenate, and others) most commonly used in dietary supplements. They are also rich in pigments, containing (by dry weight) mainly chlorophyll (0.5%–1.0%), carotenoids (0.1%–0.2%), and phycobiliproteins. The positive properties of microalgae on human health, such as prebiotics, immunomodulatory, antioxidant, anticancer and hypocholesterolemic, have been proven in many studies. The mechanism for conferring a positive effect is strongly dependent on the specific strain of microalgae and the content of bioactive substances (Hyrslova et al. 2021b).

In addition, the nutritional properties of *C. vulgaris* have led to its use as an animal feed additive. Studies conducted to date have indicated the positive effects of *C. vulgaris* on the growth of muscle mass and meat quality in poultry and pigs (Spínola et al. 2023). It has also been recognised that the inclusion in the diet of piglets of 1% Arthrospira and *C. vulgaris* , improved the overall health of the animals, providing an alternative to the use of antibiotics (Furbeyre et al. 2017). The potential impact of *C. vulgaris* on the health of livestock correlates with a higher abundance of certain specific bacterial taxa, such as *Colidextribacter*, *Oscillospira*, and *Lactobacillus*, isolated from the digestive tracts of the test animals (Martins et al. 2022). Combining algae with LAB allows the creation of a functional product containing all the essential nutrients needed for the proper and healthy functioning of the body (Ścieszka and Klewicka 2020). In the past, the effects of algal biomass on yogurt, cheese, or fermented dairy products were documented (Beheshtipour et al. 2013). The addition of algae, such as *C. vulgaris* , *C. regularis* , or Arthrospira caused a positive prebiotic effect on the viability of LAB, and also contributed to improving the nutritional quality of fermented products (Hyrslova et al. 2021b).

This study aimed to determine the effect of selected preparations containing *C. vulgaris* powder on the growth and survival of LAB exposed to adverse stress factors.

## 2 Materials and Methods

### 2.1 Strains

The present study involved the use of 30 LAB strains. Twenty‐six strains were sourced from the strain collection of the Department of Food Microbiology, Meat Technology and Chemistry at UWM Olsztyn. Four strains were obtained from the American Type Culture Collection (ATCC, United States) (Table 1). Strains were stored at −80°C in MRS broth (De Man, Rogosa and Sharpe, Merck, Germany) with a 20% addition of glycerol. The strains were revived by double passage on MRS broth and incubation at 30°C for 24 h.

**TABLE 1:** Characterisation and distribution of bacterial strains included in statistical analysis in this study.

Strain	Source of isolation	Group
*Lacticaseibacillus casei* ATCC 334	ATCC	Reference strains
*Lacticaseibacillus rhamnosus* GG (ATCC 53103)		
*Lactiplantibacillus plantarum* ATCC 8014		
*Lactobacillus acidophilus* ATCC 314		
*Lacticaseibacillus casei* Lbcs 0001	Fermented milk	Fermented products
*Lacticaseibacillus rhamnosus* Lbrm 0005	Cured sausages	
*Lactiplantibacillus plantarum* Lbp 28	Fermented beet	
*Lactiplantibacillus plantarum* Pk 1.1.	Fermented cabbage	
*Lactiplantibacillus plantarum* Rbp4	Fermented onions	
*Lactobacillus alimentarius* Lbar 1	Cured sausage	
*Lactobacillus bulgaricus* Lbbu J0001	Spontaneously fermented milk	
*Lactobacillus bulgaricus* MALUTA	Yogurt	
*Lactobacillus bulgaricus* abbbw 1003	Spontaneously fermented milk	
*Lactobacillus curvatus* Lbc 0002	Cured sausages	
*Lactobacillus sakei* Lbs 0001	Fermented leek	
*levilactobacillus brevis* ALB	Sauerkraut	
*levilactobacillus brevis* Lbbr 0001	Fermented cucumber	
*levilactobacillus brevis* Lbbv 0002	Fermented cabbage	
*Limosilactobacillus fermentum* Lf1	Bread starter	
*Lacticaseibacillus casei* Lbc 0005	Raw meat	Raw materials
*Lacticaseibacillus casei* Lbc 102		
*Lacticaseibacillus casei* Lbc 163		
*Lacticaseibacillus rhamnosus* Lbrh 0002		
*Lacticaseibacillus rhamnosus* Lbrh 0003		
*Lactobacillus alimentarius* Lbar II		
*Lactobacillus curvatus* Lbc 0001		
*Lactobacillus rhamnosus* Lbr 0001		
*Lactobacillus acidophilus*	Gastrointestinal tract of a preschool child	Gastrointestinal tract
*Lactobacillus acidophilus* Lba II		
*Lactobacillus acidophilus* Lc III		

### 2.2 Preparations

The study used two preparations containing powdered *C. vulgaris* : preparation I (dietary supplement, myVita, Poland) and preparation II (dietary supplement, Kenay, India). The recommended daily doses of the preparations, were, respectively, 2.5 g for preparation I and 3 g for preparation II. Table 2 shows the composition of preparation II. The manufacturer of preparation I did not provide a detailed product formulation.

**TABLE 2:** Composition of preparation II.

Composition in daily portion (3 g/1 tsp)	NRVs^a^	
*C. vulgaris, including*	3 g	
Vitamin A (as beta‐carotene)	2052.0 μg	260%
Vitamin B12	12.6 μg	504%
Folic acid	40.8 μg	20%
Vitamin K	35.1 μg	47%
Iron	1.5 mg	11%
Chlorophyll	78.6 mg	—
Zeaxanthin	0.8 mg	—
Lutein	4.2 mg	—

^a^ Nutrient reference values.

### 2.3 Growth

The influence of preparations containing powdered cell walls of *C. vulgaris* on the growth of LAB was compared by culturing the tested strains of LAB in modified MRS broth (enriched in *C. vulgaris* at a concentration of 3.0%). The tested concentration was selected based on the recommended daily intake of the commercial supplements used (2.5 g and 3 g for preparations I and II, respectively), ensuring physiological relevance and comparability between treatments. The abundance of the tested strains, after inoculation and 24 h of culture, was examined according to PN‐ISO 15214:2002 (Polish Committee for Standardization 2002). The control was a culture of LAB carried out in MRS broth, without the addition of algae. The cultures were carried out under anaerobic conditions, using anaerobic atmosphere generation bags (Anaerocult; Merck, Germany) in an anaerostat. The cultures were incubated at 30°C for 72 h. Results are presented as a log of colony‐forming units (CFU) per millilitre (mL).

### 2.4 Survival

The influence of preparations containing powdered cell walls of *C. vulgaris* , on the survival of LAB was compared by culturing the tested bacterial strains in modified MRS broth, enriched in *C. vulgaris* at a concentration of 3.0% and the following variants of stress factors: (a) pH brought to 3 with 1 mol/dm3 HCl (b) addition of 0.2% bile salt (BTL, Poland) (c) addition of 0.5% bile salt (BTL, Poland). The number of tested strains was examined according to PN‐ISO 15214:2002 (Polish Committee for Standardization 2002). The cultures were performed after 0 and 6 h of incubation. The control was a culture of strains of LAB conducted in stress‐enriched MRS broth, without the addition of algae. The culture was conducted under anaerobic conditions, using cartridges for generating an oxygen‐free atmosphere in an anaerostat. The cultures were incubated at 30°C for 72 h. Results are presented as log of CFU/mL.

### 2.5 Statistics

All experiments were conducted in three independent replicates. The analysed data were expressed as arithmetic means ± standard deviations (SD). For statistical analyses, the tested strains based on their source of isolation, were assigned to 4 groups: reference strains (*n* = 4), strains isolated from fermented products (*n* = 15), strains isolated from raw materials (*n* = 8), strains derived from the gastrointestinal tract (*n* = 3). The breakdown is shown in Table 1. Statistical analysis was performed using Statistica 13.0 software (StatSoft, Poland). The following tests were used: ANOVA: Kruskal‐Wallis and ANOVA: Wilcoxon at a significance level of *p* ≤ 0.05.

## 3 Results

### 3.1 Statistics

The bacterial abundances of the tested LAB strains differed significantly regardless of the applied stress factor, particularly between the control and preparations I and II (T: Wilcoxon; *p* = 0.000001). Significant differences were also observed between preparation I and II in strains incubated at pH 3 (T: Wilcoxon; *p* = 0.0052) and in the presence of 0.2% (T: Wilcoxon; *p* = 0.0023, *p* = 0.0464) and 0.5% bile salts (T: Wilcoxon; *p* = 0.0098, *p* = 0.0004) from various sources. Kruskal‐Wallis analysis confirmed significant differences among LAB strains cultured in 0.5% bile salts using preparation I (*p* = 0.0416) and preparation II (*p* = 0.1138). Multiple comparisons revealed variations within fermented products and raw materials for both preparations I (*p* = 0.0258) and II (*p* = 0.0427). Significant differences were also noted for strains incubated in 0.2% bile salts using preparation II (*p* = 0.000001), as well as between fermented products, raw materials (*p* = 0.00004), and reference strains (*p* = 0.0078). Additionally, significant differences were found between LAB strains incubated in 0.5% bile salts using preparation II (*p* = 0.0416) and between different incubation times (0–6 h) with *C. vulgaris* ‐enriched cultures in preparation I (0.2% bile salts: *p* = 0.0041; 0.5% bile salts: *p* = 0.0479).

### 3.2 Growth

The addition of *C. vulgaris* after 24 h of incubation significantly increased the abundance of LAB concerning control samples, not enriched with algae. The strains isolated from fermented products showed an increase in bacterial counts on average by 2.64 ± 0.38 log CFU/mL for preparation I, 2.58 ± 0.43 log CFU/mL for preparation II. In contrast, strains isolated from raw materials showed an average increase of 1.63 ± 1.05 log CFU/mL for formulation I, 1.68 ± 0.77 log CFU/mL for formulation II. Then, in terms of strains isolated from the gastrointestinal tract, there was an average increase of 1.51 ± 0.64 log CFU/mL for preparation I, and 1.46 ± 0.16 log CFU/mL for preparation II. The reference strains increased on average by 1.41 ± 0.37 log CFU/mL more intensively in the case of preparation I and 1.65 ± 0.71 log CFU/mL in the case of preparation II (Table 3). Detailed data on the bacterial counts of the individual strains are provided in Table S1.

**TABLE 3:** LAB abundance after exposure to powdered *C. vulgaris* preparations at 0 h and 24 h [logCFU/mL].

Group	Control	P1 (0h)	P1 (24h)	P1 (0h)	P2 (24h)
Reference strains (*n* = 4)	8.19 ± 0.15	8.51 ± 0.48	11.46 ± 0.58	10.05 ± 0.26	11.70 ± 0.79
Fermented products (*n* = 15)	7.92 ± 0.27	8.06 ± 0.34	12.13 ± 0.38	9.48 ± 0.34	12.06 ± 0.40
Raw materials (*n* = 8)	7.91 ± 0.24	7.92 ± 0.32	12.01 ± 0.67	10.38 ± 0.65	12.02 ± 0.34
Gastrointestinal tract (*n* = 3)	7.95 ± 0.17	8.25 ± 0.43	11.84 ± 0.41	10.34 ± 0.25	11.79 ± 0.28

*Note:* Values are presented as mean ± SD. Control—culture in MRS broth without the addition of *C. vulgaris* algae. P1/P2—culture in MRS broth enriched with *C. vulgaris* at a concentration of 3.0%, derived from preparation I/II.

### 3.3 Survival

Detailed data on the survival of the individual tested strains under control conditions (Table S2) and with the addition of powdered *C. vulgaris* algae from preparation I (Table S3) and preparation II (Table S4) can be found in the Supporting Information.

#### 3.3.1 pH

The initial abundance of LAB strains incubated at pH 3 varied by source. Strains from fermented products averaged 7.82 ± 0.76 log CFU/mL (control), 9.13 ± 0.72 log CFU/mL (preparation I), and 9.03 ± 0.31 log CFU/mL (preparation II). After 6 h, bacterial counts decreased by 0.36, 0.21, and 0.03 log CFU/mL, respectively. For strains from raw materials, initial counts were 8.01 ± 1.14 log CFU/mL (control), 8.89 ± 0.77 log CFU/mL (preparation I), and 9.71 ± 0.20 log CFU/mL (preparation II), with a respective decrease of 0.31, 0.25, and 0.19 log CFU/mL after 6 h. Strains from the gastrointestinal tract initially measured 8.05 ± 0.14 log CFU/mL (control), 8.89 ± 0.44 log CFU/mL (preparation I), and 9.35 ± 0.08 log CFU/mL (preparation II). After 6 h, counts changed by +0.08 (control), +0.36 (preparation I), and −0.51 log CFU/mL (preparation II). Reference strains started at 7.88 ± 0.22 log CFU/mL (control), 8.70 ± 0.43 log CFU/mL (preparation I), and 9.43 ± 0.28 log CFU/mL (preparation II). After 6 h, counts changed by +0.04 (control), +0.02 (preparation I), and −0.41 log CFU/mL (preparation II) (Table 4).

**TABLE 4:** Survival of LAB strains under low pH (pH = 3) with and without *C. vulgaris* supplementation [log CFU/mL].

Group	Control (0 h)	Control (6 h)	P1 (0 h)	P1 (6 h)	P2 (0 h)	P2 (6 h)
Reference strains (*n* = 4)	7.88 ± 0.22	7.92 ± 0.60	8.70 ± 0.43	8.72 ± 0.20	9.43 ± 0.28	9.02 ± 0.32
Fermented products (*n* = 15)	7.82 ± 0.76	7.46 ± 1.18	9.13 ± 0.72	8.92 ± 0.77	9.03 ± 0.31	9.00 ± 0.36
Raw materials (*n* = 8)	8.01 ± 1.14	7.70 ± 1.14	8.89 ± 0.77	8.64 ± 0.77	9.71 ± 0.20	9.52 ± 0.20
Gastrointestinal tract (*n* = 3)	8.05 ± 0.14	8.13 ± 0.06	8.89 ± 0.44	9.25 ± 0.39	9.35 ± 0.08	8.84 ± 0.42

*Note:* Values are presented as mean ± SD for samples incubated for 0 and 6 h at 30°C. Control—culture in MRS broth without the addition of *C. vulgaris* algae. P1/P2—culture in MRS broth enriched with *C. vulgaris* at a concentration of 3.0%, derived from preparation I/II.

### 3.4 Bile Salts

#### 3.4.1 0,2% of Bile Salts

The initial abundance of LAB strains incubated in 0.2% bile salt varied depending on the source. Strains from fermented products averaged 7.82 ± 0.76 log CFU/mL for the control, 9.06 ± 0.78 log CFU/mL for preparation I, and 9.11 ± 0.52 log CFU/mL for preparation II. After 6 h, bacterial counts decreased by 0.36, 0.19, and 0.11 log CFU/mL, respectively. Strains from raw materials had an initial abundance of 7.92 ± 0.22 log CFU/mL for the control, 8.88 ± 1.07 log CFU/mL for preparation I, and 9.55 ± 0.23 log CFU/mL for preparation II, with a respective change after 6 h of −0.10, −0.28, and + 0.17 log CFU/mL. Strains from the gastrointestinal tract initially measured 8.12 ± 0.17 log CFU/mL for the control, 8.73 ± 0.71 log CFU/mL for preparation I, and 9.51 ± 0.19 log CFU/mL for preparation II. After 6 h, bacterial counts changed by −0.15, +0.07, and −0.45 log CFU/mL, respectively. The reference strains had an initial abundance of 7.73 ± 0.22 log CFU/mL for the control, 9.30 ± 0.51 log CFU/mL for preparation I, and 9.48 ± 0.48 log CFU/mL for preparation II, with respective changes after 6 h of −0.30, −0.82, and +0.18 log CFU/mL (Table 5).

**TABLE 5:** Survival of LAB strains under bile salt stress (0.2%) with and without *C. vulgaris* supplementation [log CFU/mL].

Group	Control (0 h)	Control (6 h)	P1 (0 h)	P1 (6 h)	P2 (0 h)	P2 (6 h)
Reference strains (*n* = 4)	7.73 ± 1.31	6.89 ± 0.97	9.30 ± 0.51	8.48 ± 0.77	9.48 ± 0.48	8.96 ± 0.23
Fermented products (*n* = 15)	7.82 ± 0.76	7.46 ± 0.95	9.06 ± 0.78	8.88 ± 0.83	9.11 ± 0.52	9.01 ± 0.40
Raw materials (*n* = 8)	7.92 ± 0.22	7.82 ± 0.22	8.88 ± 1.07	8.60 ± 1.08	9.55 ± 0.23	9.73 ± 0.23
Gastrointestinal tract (*n* = 3)	8.12 ± 0.17	7.94 ± 0.20	8.73 ± 0.71	8.81 ± 0.55	9.51 ± 0.19	9.06 ± 0.19

*Note:* Values are presented as mean ± SD for samples incubated for 0 and 6 h at 30°C. Control—culture in MRS broth without the addition of *C. vulgaris* algae. P1/P2—culture in MRS broth enriched with *C. vulgaris* at a concentration of 3.0%, derived from preparation I/II.

#### 3.4.2 0.5% of Bile Salts

The initial abundance of LAB strains isolated from fermented products and incubated in 0.5% bile salt was 6.32 ± 1.26 log CFU/mL for the control, 8.60 ± 0.75 log CFU/mL for preparation I, and 8.88 ± 0.55 log CFU/mL for preparation II. After 6 h, bacterial counts decreased by 0.56, 0.45, and 0.24 log CFU/mL, respectively. Strains from raw materials had initial counts of 5.22 ± 1.24 log CFU/mL for the control, 8.00 ± 0.39 log CFU/mL for preparation I, and 9.31 ± 0.60 log CFU/mL for preparation II, with respective decreases of 0.10, 0.40, and 0.22 log CFU/mL after 6 h. Strains from the gastrointestinal tract initially measured 5.52 ± 0.46 log CFU/mL for the control, 8.00 ± 0.99 log CFU/mL for preparation I, and 9.09 ± 0.62 log CFU/mL for preparation II, with corresponding reductions of 0.20, 0.30, and 0.60 log CFU/mL after 6 h. The initial abundance of reference strains was 6.42 ± 1.46 log CFU/mL for the control, 8.58 ± 0.53 log CFU/mL for preparation I, and 8.78 ± 1.06 log CFU/mL for preparation II. After 6 h, bacterial counts decreased by 0.29 log CFU/mL for the control, 1.51 log CFU/mL for preparation I, and 0.10 log CFU/mL for preparation II (Table 6).

**TABLE 6:** Survival of LAB strains under bile salt stress (0.5%) with and without *C. vulgaris* supplementation (log CFU/mL).

Group	Control (0 h)	Control (6 h)	P1 (0 h)	P1 (6 h)	P2 (0 h)	P2 (6 h)
Reference strains (*n* = 4)	6.42 ± 1.46	5.58 ± 1.34	8.58 ± 0.53	7.06 ± 0.36	8.78 ± 1.06	8.76 ± 0.77
Fermented products (*n* = 15)	6.32 ± 1.26	5.76 ± 1.36	8.60 ± 0.75	8.15 ± 0.64	8.88 ± 0.55	8.64 ± 0.58
Raw materials (*n* = 8)	5.22 ± 1.24	5.13 ± 1.23	8.00 ± 0.39	7.60 ± 0.39	9.31 ± 0.60	9.10 ± 0.60
Gastrointestinal tract (*n* = 3)	5.52 ± 0.46	5.32 ± 1.23	8.00 ± 0.99	7.70 ± 0.67	9.09 ± 0.62	8.49 ± 0.32

*Note:* Values are presented as mean ± SD for samples incubated for 0 and 6 h at 30°C. Control—culture in MRS broth without the addition of *C. vulgaris* algae. P1/P2—culture in MRS broth enriched with *C. vulgaris* at a concentration of 3.0%, derived from preparation I/II.

## 4 Discussion

In recent years, there has been growing interest in the potential synergistic effects of *C. vulgaris* and LAB on the health benefits enjoyed by humans. In addition, the increase in consumer demand for functional fermented products has led to research into new ingredients that can be used in foods. For this reason, the use of powdered *C. vulgaris* has been investigated in the past in a soy beverage, among others, evaluating the product for its bacterial content (Ścieszka et al. 2021). The abundance of *Levilactobacillus brevis* LOCK 0944 in the soy beverage was measured during the 24‐h preparation process (4‐h fermentation at 30°C and 20‐h maturation at 20°C). After 12 h (4 h fermentation and 8 h maturation) there were statistically significant differences (*p* < 0.05; 0.5 log CFU/mL) between the control samples and samples enriched with *C. vulgaris* . The addition of *C. vulgaris* to the fermented soy beverage had a beneficial effect on the metabolic activity of *L. brevis* LOCK 0944, causing an increase in the production of L‐lactic acid. However, it is worth mentioning that after 24 h, the abundance of *L. brevis* LOCK 0944 in samples enriched with *C. vulgaris* and in the control was 9 log CFU/mL and there were no statistically significant differences in their abundance. In the present study, three other strains of *L. brevis* isolated from fermented products were tested: *L. brevis* Lbbv 0002, *L. brevis* Lbbr 0001 and *L. brevis* ALB. Their abundance increased by an average of 2.65 ± 0.24 log CFU/mL for preparation I and 2.57 ± 0.23 log CFU/mL for preparation II, compared with control samples not enriched with *C. vulgaris* . The tested strains reached abundances of 11.79–12.47 log CFU/mL in the case of preparation I and 11.73–12.36 log CFU/mL in the case of preparation II. This demonstrates the susceptibility, of all the tested strains to *C. vulgaris* . Other studies have also shown that the addition of 1.5% of *C. vulgaris* to MRS broth, accelerated the growth of *L. brevis* , shortening the growth phase of the logarithmic phase (Ścieszka et al. 2019). *L. brevis* strains are isolated from silage, fermented cabbage, and other fermented foods. They are of particular interest, as they are characterized by their status as Recognised Presumption of Safety (Qualified Presumption of Safety), consequently finding wide application in the production of fermented foods (Feyereisen et al. 2019). According to Ścieszka et al. (2019), the combination of *C. vulgaris* and *L. brevis* shows great potential in creating innovative, functional fermented products. Retrieved results in the present study, confirm the positive effect of *C. vulgaris* on the increase of the abundance of *L. brevis* strains.

Beheshtipour et al. (2012), demonstrated that the addition of microalgae significantly (*p* < 0.05) increased the survival of *Lactobacillus acidophilus* LA‐5 and *Bifidobacterium lactis* BB‐12 in yogurt, at the end of fermentation and during storage. The concentration of *C. vulgaris* at 0.1%, increased the abundance of *L. acidophilus* LA‐5 by 0.21 log CFU/mL concerning the control sample. In contrast, a concentration of 0.5% resulted in an *L. acidophilus* LA‐5 population increased by 0.47 log CFU/mL. The largest increase in *L. acidophilus* LA‐5 in the product was observed with the addition of *C. vulgaris* at a concentration of 1.0%. The product contained 0.75 log CFU/mL more tested bacteria than yogurt without added algae. In addition in yogurt samples enriched with 0.5 or 1% microalgae, the survival rate of the tested strain was higher than 10^7^ CFU/mL by the end of refrigerated storage. The authors concluded, however, that a concentration of 0.5% yields more desirable sensory qualities of the final product, and at the same time, compared to a concentration of 1%, is more economically viable. In the present study, three strains of *L. acidophilus* were tested: *L. acidophilus* Lc III, *L. acidophilus* , and *L. acidophilus* Lba II, which were isolated from the gastrointestinal tract of preschool children. The tested strains achieved 1.51 ± 0.64 CFU/mL (for preparation I) and 1.46 ± 0.16 CFU/mL (for preparation II), higher growth, than in the control, without the addition of algae. The 3.0% concentration of algae used in the present study, allowed maintaining the survival of *L. acidophilus* strains in the presence of all stress factors tested above 10^8^ CFU/mL. A product considered a probiotic must contain at least 10^6^ live bacterial cells (Sikora et al. 2020). When considering the potential economic costs, of producing health‐promoting food products enriched with algae, it would be necessary to check whether lower concentrations of preparations show a similar effectiveness. The research methodology adopted in this study mapped the pH value in the gastric juice and the concentrations of bile salts present in the intestinal juice, ranging from 0.2% to 0.5%. Importantly, the average gastrointestinal passage lasts about 3 h (Zielińska et al. 2019). The assumed stress exposure time of 6 h was chosen due to the nature of the chosen research methodology. Namely, it does not map all the stress factors to which probiotic strains are subjected during the digestion process (Jach et al. 2013; Zielińska et al. 2019). The obtained abundances of *L. acidophilus* prove that the strains studied show a high resistance to the applied stress factors.

Janczyk et al. (2009) found that the addition of *C. vulgaris* in feed significantly increased the *Lactobacillus* biodiversity in the caecum of domestic hens (*Gallus gallus domesticus*), of the Lohmann Brown breed. The most dominant bands in the DGGE image (denaturing gradient gel electrophoresis) were common to all birds and belonged to *L. gasseri* and *L. gallinarum* , species belonging to the *L. acidophilus* group (You and Kim 2020). According to the studies presented above (Janczyk et al. 2009; Beheshtipour et al. 2012), *C. vulgaris* has a positive effect on the abundance of *L. acidophilus* strains and closely related species. In the work of Abdella et al. (2023) feeds with *Lactocaseibacillus casei* ATCC 7469‐fermented soy flour and *C. vulgaris* significantly enhanced body weight, glucose tolerance, blood glucose levels, and insulin resistance in diabetic rats, demonstrating a marked improvement in their metabolic health.

A variety of preparations containing *C. vulgaris* are available on the market, varying in chemical composition, nutrient content, and production method. Other studies (Beheshtipour et al. 2012; Ścieszka and Klewicka 2020), have focused on the following: assessing the impact of *C. vulgaris* on the growth and survival of *Lactobacillus* sp., only about single‐source algae. In the present study, the effect of two independent preparations containing powdered walls of *C. vulgaris is examined*. The results obtained highlight that the effect of individual preparations containing *C. vulgaris* on the survival of LAB may be varied. This is most likely due to differences in the composition of the individual preparations. The manufacturer of preparation I, however, has not made public the detailed composition of the product, which makes a precise comparative analysis impossible. Preparation II had a higher bacterial count after 6 h of culture in the presence of all stress factors tested, except cultures of strains isolated from the gastrointestinal tract at pH = 3, but was a poorer protective matrix.

**FIGURE 1:**
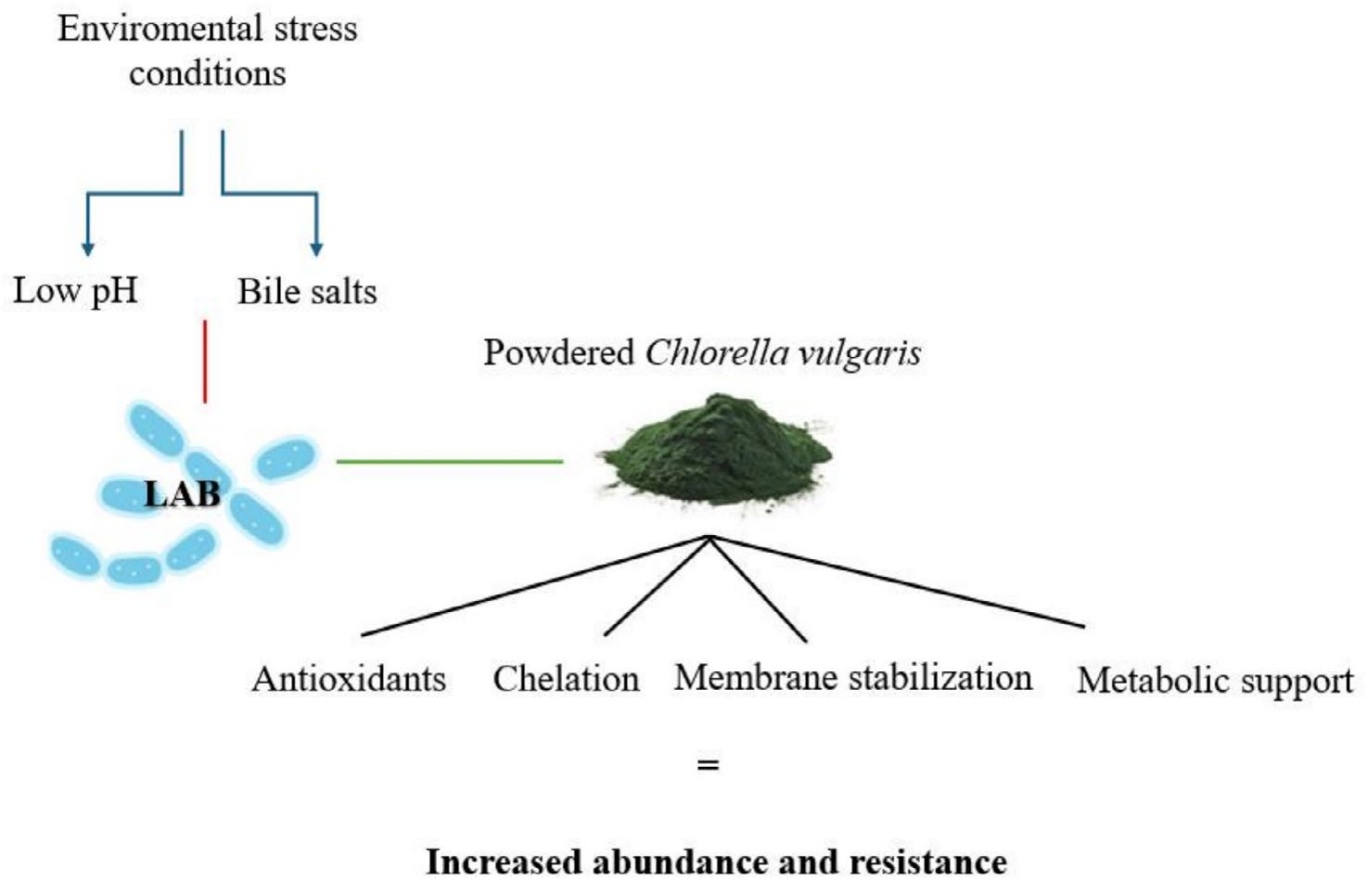
Protective mechanisms of *C. vulgaris* on LAB survival under acidic and bile stress.

Despite the promising findings, our study has limitations that should be acknowledged. One of the main constraints is the limited knowledge about the detailed chemical composition of the *C. vulgaris* preparations used, particularly Preparation I, for which the manufacturer did not disclose full specifications. As evidenced by extant literature, a number of compounds (proteins, peptides, amino acids, vitamins and chlorophyll with antioxidant activity) contained within the cells of *C. vulgaris* may exert positive effects on various bacterial taxa (Ścieszka and Klewicka 2020; Hyrslova et al. 2021a; Safari et al. 2022; Ribeiro et al. 2023). Nevertheless, β‐1,3‐glucan is most frequently referred to as the most substantial compound of a potentially prebiotic nature (Hyrslova et al. 2021a). Moreover, *C. vulgaris* can potentially influence lactic acid bacteria (LAB) by supplying vitamins, proteins, polysaccharides, and trace elements that promote their growth, metabolism, and survival, while also providing antioxidant compounds that protect against oxidative stress (Yu et al. 2023; Mendes et al. 2024). Additionally, *C. vulgaris* has the ability to chelate metal ions (e.g., Fe, Zn, Mg), which can enhance their bioavailability or, if in excess, limit growth by reducing micronutrient availability. This chelation ability is supported by studies showing biosorption and bioaccumulation of metals by *C. vulgaris* , involving cell wall components with functional groups such as amino, carboxyl, hydroxyl, and sulfate groups (Merino et al. 2019; Fitri et al. 2024). The proposed scheme of action of powdered *C. vulgaris* is presented in Figure 1. In future research, we also intend to delve deeper into the study of the transcriptome of LAB cultured in the presence of powdered cell walls of *C. vulgaris* , in order to identify which metabolic pathways are more strongly expressed in the presence of algae. Additionally, only one concentration of *C. vulgaris* (3.0%) was tested, without exploring the potential dose‐dependent response. Nevertheless, the dose that was tested corresponded to the daily dose that had been recommended by the manufacturer. This dose was the same for the two preparations, which were the most common for *C. vulgaris* enriched supplements. We also recognize the need to examine the morphological changes of LAB under stress conditions using advanced techniques such as electron microscopy. However, in a paper by Emad and Elbialy (2019), the algal biomass of *C. vulgaris* was investigated as a potential carrier for *Lacticaseibacillus casei* strains. The results of the study demonstrated that the microorganisms under investigation exhibited a tendency to adhere to each other, as evidenced by scanning electron microscope (SEM) imaging of the sample.

## 5 Conclusions

The results of the studies carried out, show the promising effect of the algae combination *C. vulgaris* and LAB. The combination of probiotic bacteria with algae may represent an innovative solution in food processing, particularly in the context of healthy lifestyle trends and increasing consumer awareness of the impact of diet on health. Further research in this area should focus on determining the optimal doses of *C. vulgaris* to achieve a beneficial effect while maintaining the desired organoleptic characteristics of the food.

## Author Contributions

**Patryk Adamski:** data curation, investigation, validation, visualisation, writing – original draft. **Lucyna Kłębukowska:** conceptualization, methodology, validation, supervision, writing – review and editing.

## Conflicts of Interest

The authors declare no conflicts of interest.

## Supporting information


**Table S1:** The growth of tested LAB strains.
**Table S2:** Survival of the studied LAB strains, cultured in modified MRS broth, without the addition of *C. vulgaris* . Results are presented as mean numer of bacteria (log CFU/mL ± SD) from three independent trials.
**Table S3:** Survival of the studied LAB strains, cultured in modified MRS broth, enriched with 3.0% *C. vulgaris* (Preparation I). Results are presented as mean numer of bacteria (log CFU/mL ± SD) from three independent trials.
**Table S4:** Survival of the studied LAB strains, cultured in modified MRS broth, enriched with 3.0% *C. vulgaris* (Preparation II). Results are presented as mean numer of bacteria (log CFU/mL ± SD) from three independent trials.

## Data Availability

The data that supports the findings of this study is available in the Supporting Information of this article.
