# Supplementary material for: The Microalga Chlorella vulgaris Supplements as a Factor Increasing the Survival of Potentially Probiotic Lactic Acid Bacteria Under Environmental Stress Conditions
Source: Environ Microbiol Rep. 2025 Nov 14;17(6):e70226. doi: 10.1111/1758-2229.70226 (PMC12616498; doi:10.1111/1758-2229.70226)
Supplement: Supplementary file 1 — Table S1: The growth of tested LAB strains. Table S2: Survival of the studied LAB strains, cultured in modified MRS broth, without the addition of C. vulgaris . Results are presented as mean numer of bacteria (log CFU/mL ± SD) from three independent trials. Table S3: Survival of the studied LAB strains, cultured in modified MRS broth, enriched with 3.0% C. vulgaris (Preparation I). Results are presented as mean numer of bacteria (log CFU/mL ± SD) from three independent trials. Table S4: Survival of the studied LAB strains, cultured in modified MRS broth, enriched with 3.0% C. vulgaris (Preparation II). Results are presented as mean numer of bacteria (log CFU/mL ± SD) from three independent trials. [file EMI4-17-e70226-s001.docx]

Table S1. The growth of tested LAB strains.

| Strain | A | B | C | D | E | F |
| --- | --- | --- | --- | --- | --- | --- |
|  |  | **log CFU/mL** | | | | |
| *L*. *plantarum* ATCC 8014 | Reference strains | 8,16 ± 0,25 | 9,18 ± 0,11 | 9,86 ± 0,12 | 10,95 ± 0,24 | 12,05 ± 0,27 |
| *L*. *rhamnosus* GG (ATCC 53103) |  | 8,45 ± 0,28 | 8,75 ± 0,27 | 9,94 ± 0,25 | 10,95 ± 0,17 | 12,06 ± 0,11 |
| *L*. *acidophilus* ATCC 314 |  | 8,06 ± 0,23 | 8,02 ± 0,29 | 10,50 ± 0,30 | 12,36 ± 0,29 | 12,35 ± 0,21 |
| *L*. *casei* ATCC 334 |  | 8,08 ± 0,28 | 8,11 ± 0,25 | 9,90 ± 0,29 | 11,59 ± 0,23 | 10,34 ± 0,27 |
| *L*. *bulgaricus* MALUTA | Fermented products | 8,41 ± 0,12 | 8,45 ± 0,19 | 9,86 ± 0,15 | 12,24 ± 0,26 | 12,39 ± 0,26 |
| *L*. *bulgaricus* abbbw 1003 |  | 8,20 ± 0,20 | 8,32 ± 0,21 | 9,15 ± 0,11 | 11,23 ± 0,35 | 12,30 ± 0,12 |
| *L*. *bulgaricus* Lbbu J0001 |  | 8,13 ± 0,34 | 8,91 ± 0,23 | 9,51 ± 0,12 | 12,47 ± 0,30 | 11,94 ± 0,18 |
| *L*. *plantarum* Rbp4 |  | 7,99 ± 0,19 | 9,50 ± 0,33 | 9,88 ± 0,16 | 11,66 ± 0,29 | 11,60 ± 0,31 |
| *L*. *plantarum* Pk 1.1. |  | 8,00 ± 0,25 | 8,10 ± 0,28 | 9,21 ± 0,21 | 11,66 ± 0,17 | 11,58 ± 0,29 |
| *L*. *plantarum* Lbp 28 |  | 7,91 ± 0,33 | 7,95 ± 0,32 | 8,89 ± 0,17 | 12,26 ± 0,17 | 12,32 ± 0,21 |
| *L*. *brevis* Lbbv 0002 |  | 7,46 ± 0,28 | 7,48 ± 0,16 | 9,27 ± 0,13 | 11,79 ± 0,33 | 11,73 ± 0,11 |
| *L*. *brevis* Lbbr 0001 |  | 7,98 ± 0,12 | 7,56 ± 0,29 | 9,48 ± 0,29 | 12,47 ± 0,17 | 12,37 ± 0,22 |
| *L*. *casei* Lbcs 0001 |  | 7,56 ± 0,33 | 7,46 ± 0,27 | 9,12 ± 0,16 | 12,00 ± 0,23 | 12,08 ± 0,30 |
| *L*. *sakei* Lbs 0001 |  | 7,90 ± 0,17 | 7,91 ± 0,30 | 9,88 ± 0,18 | 12,46 ± 0,16 | 12,28 ± 0,11 |
| *L*. *fermentum* Lf1 |  | 8,30 ± 0,16 | 8,33 ± 0,33 | 9,91 ± 0,34 | 12,46 ± 0,23 | 12,36 ± 0,32 |
| *L*. *alimentarius* Lbar 1 |  | 7,49 ± 0,24 | 7,45 ± 0,16 | 9,57 ± 0,10 | 12,47 ± 0,32 | 12,28 ± 0,24 |
| *L*. *brevis* ALB |  | 7,68 ± 0,16 | 7,77 ± 0,22 | 9,97 ± 0,31 | 12,40 ± 0,31 | 12,32 ± 0,35 |
| *L*. *rhamnosus* Lbrm 0005 |  | 7,91 ± 0,19 | 7,79 ± 0,30 | 9,48 ± 0,22 | 12,45 ± 0,20 | 12,33 ± 0,28 |
| *L*. *curvatus* Lbc 0002 |  | 7,92 ± 0,23 | 7,97  ± 0,14 | 9,10 ± 0,19 | 11,90 ± 0,28 | 11,00 ± 0,19 |
| *L*.  *rhamnosus* Lbr 0001 | Raw materials | 8,20 ± 0,32 | 8,32 ± 0,29 | 8,78 ± 0,30 | 12,39 ± 0,17 | 11,97 ± 0,22 |
| *L*. *rhamnosus* Lbrh 0003 |  | 7,68 ± 0,19 | 7,60 ± 0,21 | 10,41 ± 0,26 | 12,26 ± 0,32 | 12,39 ± 0,30 |
| *L*. *rhamnosus* Lbrh 0002 |  | 7,98 ± 0,28 | 7,96 ± 0,17 | 10,79 ± 0,19 | 12,14 ± 0,30 | 11,96 ± 0,10 |
| *L*. *curvatus* Lbc 0001 |  | 7,89 ± 0,14 | 7,38 ± 0,18 | 10,81 ± 0,18 | 10,90 ± 0,21 | 12,34 ± 0,15 |
| *L*. *casei* Lbc 163 |  | 7,68 ± 0,31 | 7,77 ± 0,26 | 10,64 ± 0,18 | 12,28 ± 0,19 | 12,08 ± 0,30 |
| *L*. *casei* Lbc 0005 |  | 7,56 ± 0,30 | 7,96 ± 0,17 | 10,65 ± 0,24 | 11,90 ± 0,22 | 11,30 ± 0,29 |
| *L*. *alimentarius* Lbar2 |  | 8,28 ± 0,23 | 8,38 ± 0,23 | 10,91 ± 0,27 | 12,87 ± 0,20 | 11,79 ± 0,28 |
| *L*. *casei* Lbc102 |  | 8,00 ± 0,20 | 8,00 ± 0,29 | 10,09 ± 0,25 | 12,38 ± 0,24 | 12,33 ± 0,12 |
| *L*. *acidophilus* Lc III | Gastrointestinal tract | 8,17 ± 0,18 | 8,07 ± 0,26 | 10,12 ± 0,19 | 12,00 ± 0,13 | 11,41 ± 0,13 |
| *L*. *acidophilus* LbaIII |  | 7,75 ± 0,21 | 7,85 ± 0,10 | 10,21 ± 0,33 | 12,26 ± 0,24 | 11,88 ± 0,14 |
| *L*. *acidophilus* Lba II |  | 7,93 ± 0,35 | 8,84 ± 0,32 | 10,68 ± 0,18 | 11,28 ± 0,34 | 12,09 ± 0,27 |

A – Source of studied strains, B - After 24h incubation in MRS broth, C – Time 0 of incubation in MRS broth in presence of *C*. *vulgaris* at a concentration of 3.0% (Preparation I), D - Time 0 of incubation in MRS broth in presence of *C*. *vulgaris* at a concentration of 3.0% (Preparation II), E - After 24h of incubation in MRS broth in presence of *C. vulgaris* at a concentration of 3.0% (Preparation I), F - After 24h of incubation in MRS broth in presence of *C*. *vulgaris* at a concentration of 3.0% (Preparation II). Results are presented as mean values from three independent trials in log CFU/mL.

Table S2. Survival of the studied LAB strains, cultured in modified MRS broth, without the addition of *C. vulgaris*. Results are presented as mean numer of bacteria (log CFU/mL ± SD) from three independent trials.

| Strain | A | B | C | D | E | F | G |
| --- | --- | --- | --- | --- | --- | --- | --- |
|  |  | **log CFU/mL** | | | | | |
| *L*. *plantarum* ATCC 8014 | Reference strains | 8,20 ± 0,27 | 8,15 ± 0,24 | 7,45 ± 0,25 | 7,93 ± 0,35 | 8,24 ± 0,18 | 5,41 ± 0,23 |
| *L*. *rhamnosus* GG (ATCC 53103) |  | 7,68 ± 0,15 | 6,48 ± 0,14 | 5,08 ± 0,26 | 8,23 ± 0,18 | 5,96 ± 0,17 | 4,60 ± 0,29 |
| *L*. *acidophilus* ATCC 314 |  | 7,67 ± 0,18 | 7,32 ± 0,33 | 5,95 ± 0,17 | 7,95 ± 0,26 | 7,38 ± 0,18 | 7,81 ± 0,29 |
| *L*. *casei* ATCC 334 |  | 7,95 ± 0,26 | 6,78 ± 0,23 | 5,00 ± 0,14 | 7,57 ± 0,15 | 5,96 ± 0,34 | 4,48 ± 0,13 |
| *L*. *bulgaricus* MALUTA | Fermented products | 7,99 ± 0,16 | 6,48 ± 0,12 | 8,01 ± 0,19 | 7,49 ± 0,28 | 5,48 ± 0,32 | 7,81 ± 0,27 |
| *L*. *bulgaricus* abbbw 1003 |  | 7,78 ± 0,19 | 6,30 ± 0,13 | 6,00 ± 0,25 | 7,76 ± 0,10 | 5,18 ± 0,23 | 6,00 ± 0,20 |
| *L*. *bulgaricus* Lbbu J0001 |  | 8,09 ± 0,26 | 7,76 ± 0,10 | 6,95 ± 0,17 | 8,47 ± 0,25 | 8,25 ± 0,28 | 5,71 ± 0,22 |
| *L*. *plantarum* Rbp4 |  | 8,30 ± 0,22 | 8,34 ± 0,13 | 6,00 ± 0,14 | 8,17 ± 0,31 | 8,41 ± 0,20 | 4,85 ± 0,31 |
| *L*. *plantarum* Pk 1.1. |  | 8,02 ± 0,30 | 7,00 ± 0,25 | 4,30 ± 0,11 | 7,94 ± 0,27 | 8,09 ± 0,22 | 4,30 ± 0,20 |
| *L*. *plantarum* Lbp 28 |  | 7,90 ± 0,16 | 7,70 ± 0,28 | 4,30 ± 0,28 | 4,60 ± 0,31 | 8,23 ± 0,31 | 5,71 ± 0,31 |
| *L*. *brevis* Lbbv 0002 |  | 7,89 ± 0,34 | 7,93 ± 0,14 | 8,00 ± 0,32 | 6,41 ± 0,15 | 8,18 ± 0,31 | 7,87 ± 0,15 |
| *L*. *brevis* Lbbr 0001 |  | 8,31 ± 0,33 | 8,15 ± 0,34 | 7,08 ± 0,24 | 8,41 ± 0,10 | 8,16 ± 0,25 | 6,30 ± 0,32 |
| *L*. *casei* Lbcs 0001 |  | 7,99 ± 0,13 | 8,39 ± 0,15 | 6,00 ± 0,16 | 7,94 ± 0,20 | 8,17 ± 0,24 | 4,85 ± 0,26 |
| *L*. *sakei* Lbs 0001 |  | 7,88 ± 0,13 | 7,66 ± 0,25 | 5,11 ± 0,10 | 7,72 ± 0,17 | 8,02 ± 0,33 | 4,00 ± 0,14 |
| *L*. *fermentum* Lf1 |  | 5,30 ± 0,26 | 7,60 ± 0,30 | 7,90 ± 0,28 | 4,78 ± 0,23 | 7,85 ± 0,11 | 8,43 ± 0,32 |
| *L*. *alimentarius* Lbar 1 |  | 8,36 ± 0,28 | 8,02 ± 0,25 | 6,00 ± 0,27 | 8,23 ± 0,20 | 8,42 ± 0,14 | 4,00 ± 0,29 |
| *L*. *brevis* ALB |  | 6,90 ± 0,26 | 4,70 ± 0,15 | 5,11 ± 0,16 | 7,85 ± 0,32 | 7,59 ± 0,18 | 4,60 ± 0,31 |
| *L*. *rhamnosus* Lbrm 0005 |  | 8,32 ± 0,19 | 7,86 ± 0,17 | 6,00 ± 0,18 | 7,98 ± 0,33 | 5,08 ± 0,30 | 6,00 ± 0,14 |
| *L*. *curvatus* Lbc 0002 |  | 8,31 ± 0,18 | 8,04 ± 0,26 | 8,08 ± 0,18 | 8,09 ± 0,27 | 7,99 ± 0,24 | 6,00 ± 0,18 |
| *L*.  *rhamnosus* Lbr 0001 | Raw materials | 8,01 ± 0,20 | 8,10 ± 0,19 | 6,70 ± 0,23 | 7,93 ± 0,32 | 7,79 ± 0,29 | 4,78 ± 0,22 |
| *L*. *rhamnosus* Lbrh 0003 |  | 8,37 ± 0,28 | 7,95 ± 0,31 | 4,78 ± 0,11 | 8,42 ± 0,18 | 7,57 ± 0,29 | 6,00 ± 0,12 |
| *L*. *rhamnosus* Lbrh 0002 |  | 7,81 ± 0,33 | 7,72 ± 0,18 | 4,00 ± 0,29 | 8,19 ± 0,28 | 7,86 ± 0,16 | 4,60 ± 0,21 |
| *L*. *curvatus* Lbc 0001 |  | 7,87 ± 0,20 | 7,88 ± 0,31 | 6,00 ± 0,17 | 7,72 ± 0,20 | 7,72 ± 0,13 | 6,00 ± 0,31 |
| *L*. *casei* Lbc 163 |  | 7,63 ± 0,25 | 7,70 ± 0,31 | 4,70 ± 0,23 | 7,70 ± 0,34 | 7,84 ± 0,16 | 4,00 ± 0,31 |
| *L*. *casei* Lbc 0005 |  | 8,20 ± 0,35 | 8,26 ± 0,14 | 4,48 ± 0,24 | 8,43 ± 0,21 | 8,33 ± 0,34 | 4,00 ± 0,15 |
| *L*. *alimentarius* Lbar2 |  | 8,29 ± 0,18 | 7,90 ± 0,27 | 4,70 ± 0,22 | 8,42 ± 0,11 | 7,57 ± 0,31 | 4,00 ± 0,19 |
| *L*. *casei* Lbc102 |  | 7,92 ± 0,33 | 7,90 ± 0,19 | 6,48 ± 0,17 | 4,78 ± 0,26 | 7,91 ± 0,23 | 7,68 ± 0,25 |
| *L*. *acidophilus* Lc III | Gastrointestinal tract | 8,16 ± 0,27 | 8,05 ± 0,26 | 5,65 ± 0,10 | 8,05 ± 0,16 | 7,69 ± 0,14 | 5,00 ± 0,31 |
| *L*. *acidophilus* LbaIII |  | 7,85 ± 0,31 | 7,96 ± 0,30 | 6,00 ± 0,23 | 8,16 ± 0,34 | 8,15 ± 0,19 | 4,00 ± 0,19 |
| *L*. *acidophilus* Lba II |  | 8,16 ± 0,12 | 8,36 ± 0,30 | 4,90 ± 0,24 | 8,20 ± 0,16 | 8,08 ± 0,11 | 6,95 ± 0,30 |

 A - Source of isolation/origin, B – Time 0 of incubation in MRS broth with pH=3, B - After 6h of incubation in MRS broth with pH=3, C - Time 0 of incubation in MRS broth enriched with 0.2% bile salts, D - After 6h of culture in MRS broth enriched with 0.2% bile salts, E – Time  0 of incubation in MRS broth enriched with 0.5% bile salts, F - After 6h of culture in MRS broth enriched with 0.5% bile salts.

Table S3. Survival of the studied LAB strains, cultured in modified MRS broth, enriched with 3,0% *C. vulgaris* (Preparation I). Results are presented as mean numer of bacteria (log CFU/mL ± SD) from three independent trials.

| Strain | A | B | C | D | E | F | G |
| --- | --- | --- | --- | --- | --- | --- | --- |
|  |  | **log CFU/mL** | | | | | |
| *L*. *plantarum* ATCC 8014 | Reference strains | 8,70 ± 0,15 | 9,90 ± 0,32 | 8,60 ± 0,17 | 7,98 ± 0,16 | 8,45 ± 0,30 | 7,08 ± 0,24 |
| *L*. *rhamnosus* GG (ATCC 53103) |  | 9,04 ± 0,17 | 9,56 ± 0,23 | 8,30 ± 0,28 | 9,60 ± 0,14 | 8,45 ± 0,16 | 7,34 ± 0,12 |
| *L*. *acidophilus* ATCC 314 |  | 8,00 ± 0,17 | 8,52 ± 0,24 | 8,00 ± 0,19 | 8,26 ± 0,12 | 8,41 ± 0,28 | 6,48 ± 0,24 |
| *L*. *casei* ATCC 334 |  | 9,08 ± 0,13 | 9,21 ± 0,28 | 9,41 ± 0,30 | 9,04 ± 0,35 | 8,61 ± 0,35 | 7,38 ± 0,18 |
| *L*. *bulgaricus* MALUTA | Fermented products | 9,56 ± 0,13 | 9,58 ± 0,20 | 9,00 ± 0,31 | 8,85 ± 0,26 | 9,59 ± 0,23 | 7,78 ± 0,15 |
| *L*. *bulgaricus* abbbw 1003 |  | 9,56 ± 0,19 | 9,90 ± 0,25 | 9,40 ± 0,29 | 10,04 ± 0,24 | 9,83 ± 0,31 | 8,95 ± 0,23 |
| *L*. *bulgaricus* Lbbu J0001 |  | 7,99 ± 0,27 | 9,78 ± 0,34 | 9,63 ± 0,27 | 7,78 ± 0,28 | 8,11 ± 0,28 | 7,51 ± 0,29 |
| *L*. *plantarum* Rbp4 |  | 8,78 ± 0,21 | 9,51 ± 0,25 | 7,85 ± 0,11 | 9,28 ± 0,21 | 9,76 ± 0,35 | 9,32 ± 0,34 |
| *L*. *plantarum* Pk 1.1. |  | 9,30 ± 0,18 | 9,45 ± 0,32 | 8,85 ± 0,17 | 9,38 ± 0,26 | 9,34 ± 0,24 | 7,41 ± 0,16 |
| *L*. *plantarum* Lbp 28 |  | 9,38 ± 0,34 | 8,51 ± 0,15 | 7,95 ± 0,27 | 9,30 ± 0,29 | 9,45 ± 0,32 | 8,85 ± 0,22 |
| *L*. *brevis* Lbbv 0002 |  | 8,70 ± 0,18 | 9,08 ± 0,30 | 8,78 ± 0,10 | 7,83 ± 0,13 | 7,56 ± 0,30 | 7,78 ± 0,34 |
| *L*. *brevis* Lbbr 0001 |  | 10,08 ± 0,35 | 9,40 ± 0,16 | 9,08 ± 0,32 | 7,95 ± 0,18 | 8,08 ± 0,11 | 7,48 ± 0,29 |
| *L*. *casei* Lbcs 0001 |  | 9,74 ± 0,27 | 9,48 ± 0,32 | 9,41 ± 0,27 | 9,48 ± 0,29 | 9,32 ± 0,12 | 7,70 ± 0,17 |
| *L*. *sakei* Lbs 0001 |  | 9,83 ± 0,27 | 9,60 ± 0,22 | 9,40 ± 0,23 | 9,54 ± 0,22 | 9,40 ± 0,16 | 8,40 ± 0,31 |
| *L*. *fermentum* Lf1 |  | 8,60 ± 0,30 | 8,48 ± 0,10 | 7,40 ± 0,13 | 9,62 ± 0,25 | 8,32 ± 0,21 | 9,26 ± 0,28 |
| *L*. *alimentarius* Lbar 1 |  | 9,56 ± 0,17 | 9,64 ± 0,24 | 8,30 ± 0,26 | 9,40 ± 0,30 | 9,64 ± 0,10 | 8,48 ± 0,15 |
| *L*. *brevis* ALB |  | 9,30 ± 0,29 | 8,85 ± 0,33 | 8,48 ± 0,20 | 9,51 ± 0,26 | 9,36 ± 0,14 | 7,77 ± 0,33 |
| *L*. *rhamnosus* Lbrm 0005 |  | 9,26 ± 0,25 | 7,26 ± 0,25 | 7,08 ± 0,33 | 8,14 ± 0,21 | 8,00 ± 0,23 | 7,89 ± 0,23 |
| *L*. *curvatus* Lbc 0002 |  | 7,30 ± 0,13 | 7,48 ± 0,25 | 8,48 ± 0,35 | 7,74 ± 0,12 | 7,38 ± 0,11 | 7,70 ± 0,26 |
| *L*.  *rhamnosus* Lbr 0001 | Raw materials | 9,67 ± 0,13 | 9,41 ± 0,18 | 8,95 ± 0,10 | 9,18 ± 0,18 | 6,48 ± 0,14 | 7,46 ± 0,17 |
| *L*. *rhamnosus* Lbrh 0003 |  | 10,45 ± 0,20 | 9,77 ± 0,32 | 8,85 ± 0,11 | 8,16 ± 0,17 | 8,45 ± 0,21 | 7,81 ± 0,27 |
| *L*. *rhamnosus* Lbrh 0002 |  | 8,95 ± 0,12 | 8,03 ± 0,14 | 7,43 ± 0,22 | 9,40 ± 0,13 | 9,64 ± 0,21 | 8,48 ± 0,32 |
| *L*. *curvatus* Lbc 0001 |  | 7,30 ± 0,25 | 7,48 ± 0,25 | 8,48 ± 0,20 | 7,74 ± 0,27 | 7,38 ± 0,28 | 7,70 ± 0,13 |
| *L*. *casei* Lbc 163 |  | 9,34 ± 0,23 | 8,70 ± 0,20 | 7,60 ± 0,15 | 9,00 ± 0,27 | 8,95 ± 0,27 | 7,08 ± 0,18 |
| *L*. *casei* Lbc 0005 |  | 9,32 ± 0,13 | 9,51 ± 0,27 | 7,53 ± 0,31 | 7,40 ± 0,10 | 9,40 ± 0,11 | 7,40 ± 0,25 |
| *L*. *alimentarius* Lbar2 |  | 8,48 ± 0,21 | 9,36 ± 0,17 | 7,70 ± 0,16 | 8,60 ± 0,31 | 9,76 ± 0,33 | 7,48 ± 0,24 |
| *L*. *casei* Lbc102 |  | 7,67 ± 0,31 | 8,85 ± 0,30 | 7,46 ± 0,32 | 9,73 ± 0,15 | 8,78 ± 0,17 | 7,40 ± 0,12 |
| *L*. *acidophilus* Lc III | Gastrointestinal tract | 9,00 ± 0,32 | 9,04 ± 0,31 | 8,78 ± 0,24 | 9,81 ± 0,31 | 8,15 ± 0,20 | 7,00 ± 0,21 |
| *L*. *acidophilus* LbaIII |  | 8,30 ± 0,35 | 7,75 ± 0,22 | 6,60 ± 0,21 | 9,00 ± 0,22 | 9,49 ± 0,26 | 8,60 ± 0,17 |
| *L*. *acidophilus* Lba II |  | 9,36 ± 0,17 | 9,41 ± 0,16 | 8,60 ± 0,12 | 8,95 ± 0,33 | 8,78 ± 0,29 | 7,49 ± 0,31 |

A - Source of isolation/origin, B – Time 0 of incubation in MRS broth with pH=3, B - After 6h of incubation in MRS broth with pH=3, C - Time 0 of incubation in MRS broth enriched with 0.2% bile salts, D - After 6h of culture in MRS broth enriched with 0.2% bile salts, E – Time  0 of incubation in MRS broth enriched with 0.5% bile salts, F - After 6h of incubation in MRS broth enriched with 0.5% bile salts.

Table S4. Survival of the studied LAB strains, cultured in modified MRS broth, enriched with 3,0% *C. vulgaris* (Preparation II). Results are presented as mean numer of bacteria (log CFU/mL ± SD) from three independent trials.

| Strain | A | B | C | D | E | F | G |
| --- | --- | --- | --- | --- | --- | --- | --- |
|  |  | **log CFU/mL** | | | | | |
| *L*. *plantarum* ATCC 8014 | Reference strains | 9,04 ± 0,30 | 7,70 ± 0,17 | 9,66 ± 0,25 | 8,95 ± 0,26 | 8,75 ± 0,17 | 9,94 ± 0,29 |
| *L*. *rhamnosus* GG (ATCC 53103) |  | 9,20 ± 0,28 | 9,73 ± 0,32 | 9,45 ± 0,23 | 8,95 ± 0,25 | 9,30 ± 0,30 | 8,30 ± 0,20 |
| *L*. *acidophilus* ATCC 314 |  | 9,40 ± 0,31 | 9,95 ± 0,17 | 8,60 ± 0,30 | 9,08 ± 0,29 | 8,75 ± 0,26 | 7,90 ± 0,17 |
| *L*. *casei* ATCC 334 |  | 10,29 ± 0,20 | 7,74 ± 0,13 | 7,72 ± 0,34 | 9,30 ± 0,17 | 9,03 ± 0,17 | 8,90 ± 0,15 |
| *L*. *bulgaricus* MALUTA | Fermented products | 9,14 ± 0,12 | 8,98 ± 0,16 | 9,27 ± 0,31 | 9,01 ± 0,20 | 9,01 ± 0,18 | 8,63 ± 0,17 |
| *L*. *bulgaricus* abbbw 1003 |  | 9,39 ± 0,13 | 9,58 ± 0,18 | 9,28 ± 0,23 | 9,30 ± 0,32 | 9,50 ± 0,22 | 9,18 ± 0,30 |
| *L*. *bulgaricus* Lbbu J0001 |  | 8,44 ± 0,15 | 9,43 ± 0,12 | 9,36 ± 0,20 | 8,54 ± 0,27 | 8,45 ± 0,29 | 8,38 ± 0,25 |
| *L*. *plantarum* Rbp4 |  | 8,91 ± 0,17 | 9,30 ± 0,19 | 8,35 ± 0,19 | 9,18 ± 0,21 | 9,42 ± 0,14 | 9,03 ± 0,24 |
| *L*. *plantarum* Pk 1.1. |  | 9,19 ± 0,14 | 9,27 ± 0,11 | 8,95 ± 0,28 | 9,23 ± 0,32 | 9,42 ± 0,19 | 9,20 ± 0,32 |
| *L*. *plantarum* Lbp 28 |  | 8,86 ± 0,26 | 8,98 ± 0,24 | 9,33 ± 0,18 | 9,05 ± 0,11 | 9,07 ± 0,11 | 8,95 ± 0,27 |
| *L*. *brevis* Lbbv 0002 |  | 8,74 ± 0,21 | 9,17 ± 0,33 | 9,33 ± 0,25 | 9,05 ± 0,14 | 9,07 ± 0,15 | 8,95 ± 0,33 |
| *L*. *brevis* Lbbr 0001 |  | 9,09 ± 0,33 | 9,15 ± 0,23 | 8,17 ± 0,16 | 8,98 ± 0,19 | 9,21 ± 0,24 | 7,98 ± 0,17 |
| *L*. *casei* Lbcs 0001 |  | 9,28 ± 0,15 | 9,05 ± 0,15 | 9,18 ± 0,28 | 9,42 ± 0,22 | 9,39 ± 0,20 | 9,13 ± 0,27 |
| *L*. *sakei* Lbs 0001 |  | 9,36 ± 0,35 | 8,65 ± 0,29 | 8,63 ± 0,14 | 8,63 ± 0,17 | 9,19 ± 0,13 | 9,41 ± 0,26 |
| *L*. *fermentum* Lf1 |  | 8,91 ± 0,10 | 9,34 ± 0,13 | 7,84 ± 0,26 | 8,50 ± 0,11 | 8,64 ± 0,24 | 7,62 ± 0,19 |
| *L*. *alimentarius* Lbar 1 |  | 8,46 ± 0,15 | 8,41 ± 0,17 | 8,98 ± 0,26 | 8,63 ± 0,10 | 8,65 ± 0,13 | 8,54 ± 0,24 |
| *L*. *brevis* ALB |  | 9,52 ± 0,19 | 10,38 ± 0,28 | 9,57 ± 0,30 | 9,68 ± 0,13 | 9,36 ± 0,31 | 7,36 ± 0,15 |
| *L*. *rhamnosus* Lbrm 0005 |  | 9,17 ± 0,35 | 7,96 ± 0,33 | 7,84 ± 0,32 | 8,54 ± 0,12 | 8,45 ± 0,34 | 8,38 ± 0,24 |
| *L*. *curvatus* Lbc 0002 |  | 9,01 ± 0,34 | 9,07 ± 0,24 | 9,11 ± 0,19 | 9,35 ± 0,22 | 8,26 ± 0,33 | 8,91 ± 0,28 |
| *L*.  *rhamnosus* Lbr 0001 | Raw materials | 9,71 ± 0,24 | 9,43 ± 0,21 | 9,40 ± 0,13 | 9,70 ± 0,29 | 9,25 ± 0,14 | 9,33 ± 0,19 |
| *L*. *rhamnosus* Lbrh 0003 |  | 9,69 ± 0,24 | 9,70 ± 0,13 | 9,85 ± 0,21 | 9,52 ± 0,23 | 9,73 ± 0,16 | 9,98 ± 0,18 |
| *L*. *rhamnosus* Lbrh 0002 |  | 9,54 ± 0,25 | 9,43 ± 0,28 | 9,35 ± 0,21 | 9,28 ± 0,16 | 9,56 ± 0,17 | 9,19 ± 0,10 |
| *L*. *curvatus* Lbc 0001 |  | 9,60 ± 0,15 | 9,64 ± 0,12 | 9,28 ± 0,27 | 9,61 ± 0,10 | 9,79 ± 0,29 | 9,50 ± 0,14 |
| *L*. *casei* Lbc 163 |  | 10,16 ± 0,20 | 9,70 ± 0,18 | 9,85 ± 0,17 | 9,89 ± 0,11 | 9,89 ± 0,29 | 9,35 ± 0,24 |
| *L*. *casei* Lbc 0005 |  | 9,60 ± 0,21 | 9,65 ± 0,31 | 8,79 ± 0,27 | 9,50 ± 0,13 | 9,70 ± 0,27 | 8,62 ± 0,15 |
| *L*. *alimentarius* Lbar2 |  | 9,43 ± 0,24 | 9,38 ± 0,30 | 8,85 ± 0,30 | 9,30 ± 0,33 | 9,81 ± 0,26 | 7,85 ± 0,15 |
| *L*. *casei* Lbc102 |  | 9,96 ± 0,17 | 9,51 ± 0,15 | 9,15 ± 0,21 | 9,34 ± 0,14 | 10,08 ± 0,23 | 8,95 ± 0,13 |
| *L*. *acidophilus* Lc III | Gastrointestinal tract | 9,24 ± 0,27 | 9,47 ± 0,17 | 8,25 ± 0,23 | 8,50 ± 0,29 | 8,81 ± 0,11 | 8,16 ± 0,25 |
| *L*. *acidophilus* LbaIII |  | 9,43 ± 0,31 | 9,43 ± 0,34 | 9,28 ± 0,31 | 9,43 ± 0,12 | 9,28 ± 0,20 | 8,39 ± 0,15 |
| *L*. *acidophilus* Lba II |  | 9,38 ± 0,30 | 9,63 ± 0,23 | 9,73 ± 0,33 | 8,60 ± 0,32 | 9,09 ± 0,23 | 8,93 ± 0,22 |

A - Source of isolation/origin, B – Time 0 of incubation in MRS broth with pH=3, B - After 6h of incubation in MRS broth with pH=3, C - Time 0 of incubation in MRS broth enriched with 0.2% bile salts, D - After 6h of culture in MRS broth enriched with 0.2% bile salts, E – Time  0 of incubation in MRS broth enriched with 0.5% bile salts, F - After 6h of incubation in MRS broth enriched with 0.5% bile salts.
